# EGF-Induced Expansion of Migratory Cells in the Rostral Migratory Stream

**DOI:** 10.1371/journal.pone.0046380

**Published:** 2012-09-28

**Authors:** Olle R. Lindberg, Åsa Persson, Anke Brederlau, Aidin Shabro, Hans Georg Kuhn

**Affiliations:** Center for Brain Repair and Rehabilitation, Institute of Neuroscience and Physiology, Sahlgrenska Academy, University of Gothenburg, Gothenburg, Sweden; Universitätsklinikum Carl Gustav Carus an der Technischen Universität Dresden, Germany

## Abstract

The presence of neural stem cells in the adult brain is currently widely accepted and efforts are made to harness the regenerative potential of these cells. The dentate gyrus of the hippocampal formation, and the subventricular zone (SVZ) of the anterior lateral ventricles, are considered the main loci of adult neurogenesis. The rostral migratory stream (RMS) is the structure funneling SVZ progenitor cells through the forebrain to their final destination in the olfactory bulb. Moreover, extensive proliferation occurs in the RMS. Some evidence suggest the presence of stem cells in the RMS, but these cells are few and possibly of limited differentiation potential. We have recently demonstrated the specific expression of the cytoskeleton linker protein radixin in neuroblasts in the RMS and in oligodendrocyte progenitors throughout the brain. These cell populations are greatly altered after intracerebroventricular infusion of epidermal growth factor (EGF). In the current study we investigate the effect of EGF infusion on the rat RMS. We describe a specific increase of radixin^+^/Olig2^+^ cells in the RMS. Negative for NG2 and CNPase, these radixin^+^/Olig2^+^ cells are distinct from typical oligodendrocyte progenitors. The expanded Olig2^+^ population responds rapidly to EGF and proliferates after only 24 hours along the entire RMS, suggesting local activation by EGF throughout the RMS rather than migration from the SVZ. In addition, the radixin^+^/Olig2^+^ progenitors assemble in chains *in vivo* and migrate in chains in explant cultures, suggesting that they possess migratory properties within the RMS. In summary, these results provide insight into the adaptive capacity of the RMS and point to an additional stem cell source for future brain repair strategies.

## Introduction

Neurogenesis persists in two distinct niches in the adult brain; the dentate gyrus of the hippocampal formation and the subventricular zone (SVZ) of the forebrain [1]. The SVZ supplies new cells to the olfactory bulb (OB) via the rostral migratory stream (RMS) which stretches across the entire forebrain [2]. The RMS is derived from the wall of the collapsed embryonic olfactory ventricle and is structurally more similar to the SVZ than to the brain tissue it passes through [3], [4]. The unique composition of the RMS includes cell type-specific expression of proteins, such as polysialylated neural cell adhesion molecule (PSA-NCAM), integrins and tenascin C, which promote cell-cell and cell-matrix interactions in the RMS [5]–[7]. In addition, microtubule-associated proteins, such as Doublecortin (DCX), are indispensable for maintaining a bipolar morphology and for nuclear translocation during neuroblast migration [8], [9]. The transduction of extracellular signals to intracellular cytoskeletal responses is important during RMS migration and may be mediated by the cytoskeleton linker proteins of the ERM (ezrin, radixin, and moesin) family. The ERM proteins regulate cellular morphology, including axonal outgrowth and motility, through their ability to connect the actin cytoskeleton to transmembrane protein complexes, such as intercellular adhesion molecules and integrins [10]–[13]. We have recently characterized the expression of ERM proteins in the adult brain [14]. In the RMS, radixin was expressed in neuroblasts and oligodendrocyte progenitors. Evidence is emerging that the RMS is more than a migratory pathway, giving rise to new cells along its entire stretch. Apart from proliferating migratory neuroblasts, the RMS has been suggested to house multipotent neural stem cells, generating both neurons and glia [15], [16]. Whether the RMS is a mere tube for channeling cells or a neurogenic niche as active as the SVZ makes a significant difference for putative therapeutic paradigms using the RMS as a stem cell reservoir. Epidermal growth factor (EGF) is a mitogen involved in regulating neural stem cell proliferation and fate determination [17]–[19]. In the SVZ, increased EGFR signaling reduces neurogenesis in favor of oligodendrogenesis [20]. The EGF induced oligodendrocyte progenitors are highly migratory and able to remyelinate injured white matter [21]. Commitment to the oligodendrocyte lineage is thought to reduce neuroblast differentiation of neural stem cells, leading to a decrease of migrating neuroblasts in the SVZ and the RMS [22], [23]. Previous studies showed reduced numbers of neuroblasts in the SVZ and RMS after EGF treatment; however, to our knowledge, a detailed description of the RMS following EGF treatment has not yet been performed. Considering that both neuroblasts and oligodendrocyte progenitors of the neurogenic niches express radixin, we aimed at tracking changes in radixin-expressing cells in the RMS after intraventricular infusion of EGF. Our results show a substantial increase of a migratory oligodendrocyte progenitor cell population in the RMS, expressing radixin, in response to EGF stimulation.

## Materials and Methods

### Ethics statement

All animal work was conducted according to European and Swedish animal welfare regulations and approved by the Gothenburg committee of the Swedish Animal Welfare Agency (application no. 145/10 and 32/11).

### Surgery and tissue preparation

Male Wistar rats, 7–8 weeks old, with an average weight of 221±14 grams were used in the study. All surgeries were performed under ketamine (33 mg/mL Ketalar, Pfizer) and xylazine (6.67 mg/mL Rompun, Bayer Healthcare AG) anesthesia, and all efforts were made to minimize suffering. The animals were divided into six groups receiving either vehicle (artificial cerebrospinal fluid, aCSF) or EGF (360 ng/day), for 1, 7, or 14 days. The surgeries were performed as described in Lindberg et al [19]. Briefly, the minipump (Model 1002; Alzet-Durect) and infusion cannula (Brain Infusion Kit 2; Alzet-Durect) were filled and primed by incubation in sterile PBS at 37°C for 24 h, to ensure no lag time in delivery after implantation. The pumps were inserted intracerebroventricularly using a stereotax (David Kopf and Stoelting Co). All animals received three intraperitoneal injections of 50 mg/kg of bromodeoxyuridine (BrdU) during the last 24 hours before perfusion. At the end of the EGF infusion period, animals were sedated using an overdose of pentobarbital and transcardially perfused with sterile 0.9% NaCl followed by phosphate-buffered (0.1 M pH 7.4) 4% paraformaldehyde (PFA). Following perfusion, brains were removed and postfixed in 4% PFA for 24 hours and prepared for cryosectioning by incubation in phosphate-buffered (0.1 M pH 7.4) 30% sucrose for at least 3 days. All brains used for cellular quantification were cryosectioned coronally in 40 µm thick serial sections using a sliding microtome and collected as serial sections (1 in 12 series with 480 µm distance between sections). Brains used for RMS composition analysis were sectioned sagittaly at 25 µm thickness. Sections were stored at 4°C in a cryoprotective solution (glycerol, ethylene glycol, and 0.1 M phosphate buffer, pH 7.4, 3∶3∶4 by volume).

### Gene expression analysis

Quantitative polymerase chain reaction was performed on micro-dissected lateral ventricle wall and olfactory bulb tissue bilaterally and processed according to Lindberg et al [19]. Primer sequences were generated using NCBI primer-BLAST (http://www.ncbi.nlm.nih.gov/tools/primer-blast/) and Primer express software (Applied Biosystems) and synthesized by Eurofins MWG Operon (Ebersberg, Germany). Primer sequences were designed with a melting temperature of 60°C spanning an intron site when possible and the efficiency of all primers was tested using a dilution curve. Sequences used: AAAGCCCAGGCCCAATGCGC (*DCX*, forward) and ACAAGTCCTTGTGCTTCCGCAGAC (*DCX*, reverse), TGTGATGGACTCCGGAGACGGG (*β-actin*, forward), TGTAGCCACGCTCGGTCAGGAT (*β-actin*, reverse), AACCCATCACCATCTTCCAGGAGCG (*GAPDH*, forward), ACATACTCAGCACCAGCATCACCCC (*GAPDH*, reverse), CCTACCACAGCGTGTTTTGGA (*Radixin* forward), TCCCCCTGTGTTCTTCATGC (*Radixin* reverse). For both primers we used an initial cycle of 15 min at 95°C, followed by repeated cycles of 94, 55, and 72°C (40 in total). A final continuous fluorescence reading at descending temperatures (melting curve) starting at 95°C was acquired to ensure proper primer functionality and to exclude primer-dimer formation. Fold-changes were calculated using C_q_ and the ΔΔC_q_ method normalizing against two reference genes (*GAPDH* and *β-actin*) according to Vandesompele et al. [24], described in detail in Lindberg et al. [19].

### Immunohistochemistry

The sections were washed in Tris-buffered saline (TBS). For all immunostainings where rabbit α-radixin or mouse α-radixin were used, the washing step was followed by antigen retrieval in 0.01 M sodium citrate pH6 at 97° for 20 minutes. Sections used for BrdU immunofluorescence were treated with 2 M HCl for 30 minutes at room temperature or 37°C followed by neutralization in 0.1 M borate buffer. All sections were blocked for 1 h in TBS with 3% donkey serum and 0.1% Triton-X (0.2% for radixin antibodies) at room temperature, prior to primary antibody incubation. Primary antibody incubation (for antibodies and concentrations see Table 1.) was performed at 4°C for 24–72 h followed by washing in TBS. Secondary antibody incubation was performed at room temperature for 2 h. The following secondary antibodies were used; CF555 donkey (Dk) α-rabbit IgG, CF555 Dk α-goat IgG, CF633 Dk α-goat IgG, CF488 Dk α-mouse IgG (Biotium), Alexa Dk α-rabbit 488, Alexa Dk α-rat 488 (Molecular Probes). ToPro3 or YoPro1 were used as nuclear stains (Molecular Probes). After secondary antibody incubation, the sections were washed in TBS and mounted using ProLong Gold with DAPI (Molecular Probes).

**Table 1:** Primary antibodies used for immunohistochemistry and immunocytochemistry.

Antibody	Host	Concentration	Company
βIII-tubulin	Mouse	1∶1000	Sigma
BrdU	Rat	1∶500	Serotech
CNPase	Mouse	1∶100	Abcam
DCX	Goat	1∶1000	Santa Cruz
GFAP	Mouse	1∶1000	Millipore
NG2	Mouse	1∶500	Millipore
Olig2	Goat	1∶500	R&D Systems
pERM	Rabbit	1∶500	Cell Signaling
Radixin	Rabbit	1∶250	Abcam
Radixin	Mouse	1∶250	Abnova
Sox2	Rabbit	1∶200	Millipore

### Confocal microscopy and quantification

For all quantifications, coronal sections were used. Immunofluorescence was visualized using a Leica SP2 scanning confocal microscope (Leica Microsystems) and a 63× objective. Each fluorochrome was recorded individually in sequential scan mode to avoid channel mixing. Images were acquired from the RMS (anterioposterior coordinates 11.35 mm to 14.50 mm from interaural line) in z-stacks of 2 µm increments. For radixin/DCX and radixin/Olig2 quantifications, cell nuclei were selected at random in the RMS, using ToPro3 and selected cells analyzed for either single or double immunoreactivity. BrdU/radixin and BrdU/Olig2 colabeling was analyzed by selecting at least 250 BrdU positive cells per animal for colocalization of immunosignals. Sox2^high/low^ expression in the expanded Olig2 population of the EGF-treated RMS was quantified at 3 to 4 locations in a total of about 75 Olig2^+^ cells per animal after 7 days of EGF infusion. Double labeling was assumed when cells exhibited direct colocalization or when nucleus and cytosol or processes from the same cell were individually labeled.

### SVZ explant cultures

Animals were anesthetized using isofluorane and decapitated. The brains were dissected and placed in ice cold HBBS until further processing. One-millimeter coronal slices were cut between anterioposterior coordinates Bregma −0.5–2.5 using a coronal brain matrix. The slices were kept on ice while the lateral ventricle walls were removed and cut into 50–200 µm diameter pieces. The tissue pieces were resuspended in Neurobasal A medium (Invitrogen) and mixed 1∶3 with Matrigel (BD Bioscience). 15 µl of the Matrigel-tissue mixture was dispensed in 8-well chamber slides (BD Bioscience), followed by 10 minutes polymerization at 37°C. Explants were grown in Neurobasal A medium, supplemented with B27 and Glutamax, PenStrep (All from Invitrogen). The explants cultures were kept at 37°C in 5% O_2_ and 1% CO_2_ for 72 h. At the end of the experiment the explants were fixed in 4% PFA for 20 minutes. Immunocytochemistry was performed in the chamber slides. Following three 15-minute washes in PBS unspecific antibody binding was blocked by incubation with 3% donkey serum and 0.2% Triton-X in PBS for three hours at room temperature. Explants were then incubated with primary antibodies (see Table 1) for 72 h at 4°C, followed by three 15-minute washes in PBS and secondary antibody incubation for 2 hours at room temperature. After additional washing steps, the explant cultures were coverslipped using Prolong Gold with DAPI (Molecular Probes) and analyzed by confocal microscopy.

### Statistics

In all comparisons, except radixin/BrdU cell ratios, the 2-tailed Student's *t*-test was employed. The Mann-Whitney U-test was used for the comparison of radixin/BrdU ratios. All statistical calculations and graphical visualizations were performed in GraphPad Prism 5 (GraphPad Software). All error bars represent standard error of the mean (SEM). Cell numbers and animal weights are presented as mean±SE. Differences of p<0.05 were considered statistically significant (*).

## Results

### Morphological changes in the RMS as a result of EGF treatment

**Figure 1:**
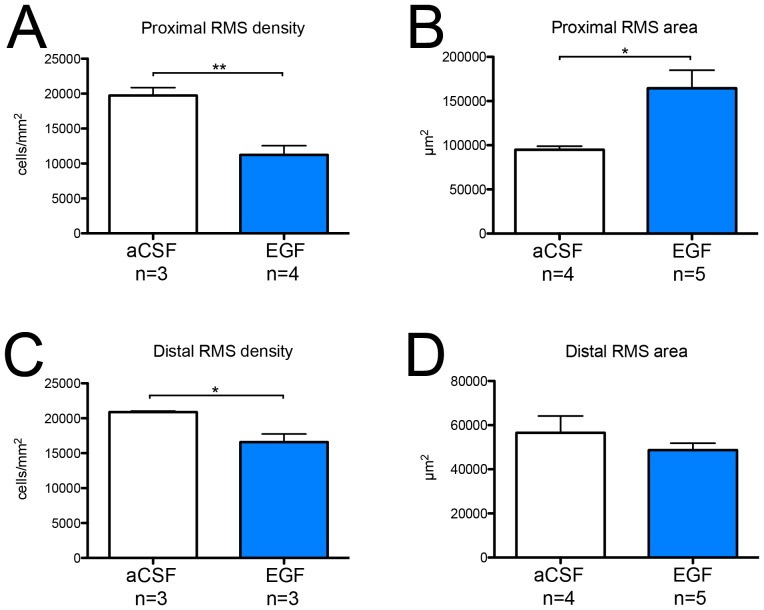
Cell density and size of the rostral migratory stream after EGF infusion. To assess EGF-induced structural changes in the rostral migratory stream (RMS) cell density and cross-sectional area were quantified at a proximal and a distal position relative to the SVZ. (**A, C**) Cell density was determined by counting cell nuclei and expressed as the number of cells per mm^2^ ± SEM. (**B, D**) The size of the RMS was determined by tracing the border of the RMS on coronal sections. Data are presented as average cross-sectional area per section expressed in mm^2^ ±SEM. For both analyses 1–4 coronal sections per animal were used. Statistical significance was assumed at * p<0.05 and ** p<0.01 using Student's t-test.

To assess structural changes in the RMS induced by EGF infusion we quantified the area and cell density at two locations along the RMS; one proximal to the SVZ (bregma +2.50 mm) and a distal location closer to the olfactory bulb (bregma +3.00 mm). In the proximal RMS we observed a reduced cell density along with an increase in RMS cross-sectional area after 7 days of EGF infusion (Figure 1A and B). The boundaries of the proximal RMS stimulated by EGF were more diffuse (data not shown), possibly due to a dispersion of neuroblasts and other progenitor cells. In the distal RMS the cell density was slightly reduced in the EGF-treated RMS (Figure 1C); however, the RMS area was unchanged compared to control distal RMS (Figure 1D).

### DCX expression is reduced in the subventricular zone and olfactory bulb following EGF infusion

**Figure 2:**
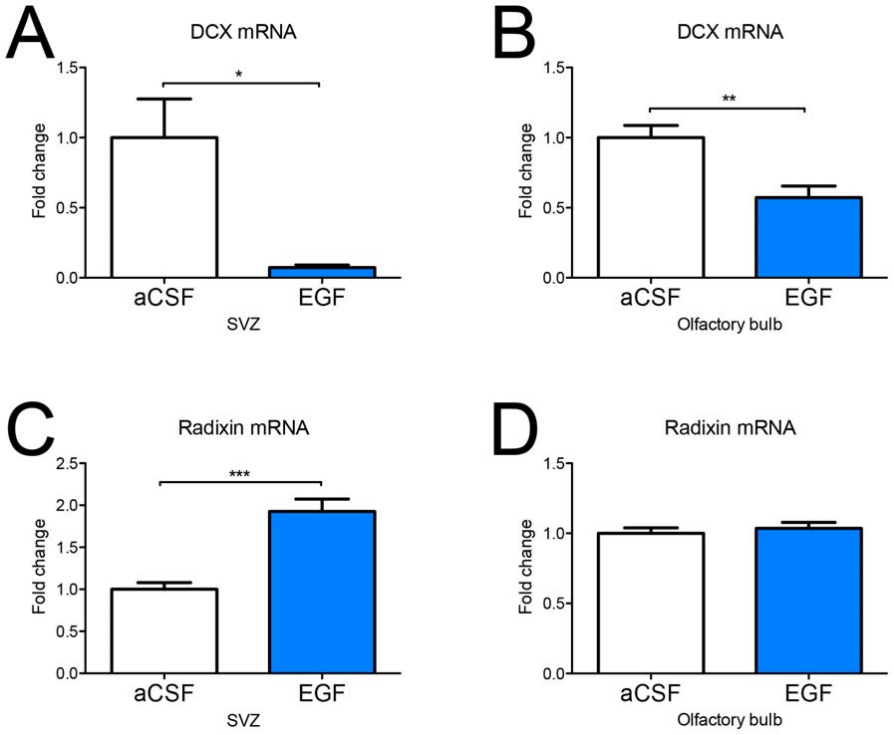
mRNA expression of *doublecortin* and *radixin* in the SVZ and olfactory bulb after EGF infusion. mRNA expression of the neuroblast-associated marker *doublecortin* (*DCX*) and *radixin* in the subventricular zone (SVZ) and olfactory bulb (OB) after 14 days of EGF infusion were determined by qPCR. Relative mRNA levels of *DCX* (**A, B**) and *radixin* (**C, D**) are expressed as fold-change compared to control ± SEM using the Delta-Delta Ct method. Statistical significance was assumed at * p<0.05, ** p<0.01 and *** p<0.001 using Student's t-test. n = 5 animals for the aCSF and the EGF group.

**Figure 3:**
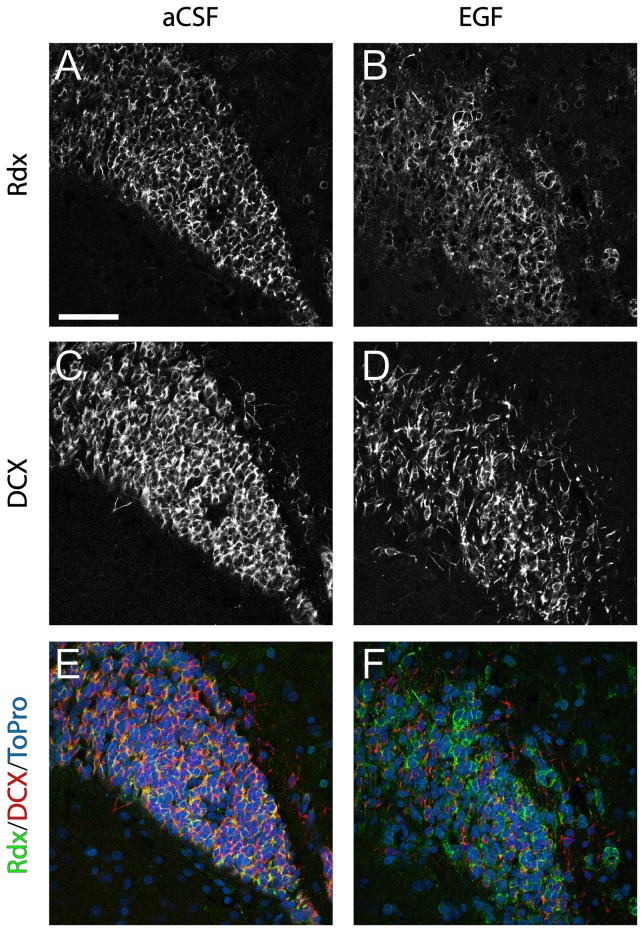
Radixin and doublecortin protein expression in the rostral migratory stream. Immunofluorescence staining illustrating radixin (Rdx) and doublecortin (DCX) expression in coronal sections of the rostral migratory stream (RMS) after 7 days of aCSF or EGF infusion. (**A, B**) Color separation for radixin, (**C, D**) color separation for DCX, (**E, F**) color composite images of radixin (green), DCX (red) and the nuclear counterstain ToPro3 (blue). The high degree of DCX/radixin colabeling in controls (yellow labeling in E) is notably reduced in EGF infused animals. Scale bar in A = 50 µm.

We previously showed co-expression of DCX and radixin in neuroblasts of the adult mouse SVZ and RMS [14]. One of the most profound effects of intracerebroventricular infusion of EGF is the reduction of neuroblasts in the SVZ and RMS [17]. Using quantitative PCR we detected a strong and persistent downregulation of *DCX* mRNA expression in the SVZ (Figure 2A) and the OB (Figure 2B) following 14 days of EGF infusion. *DCX* mRNA was downregulated to 10% of the control level in the EGF-treated SVZ and to 50% of control level in the OB. Immunofluorescence analysis of DCX protein expression after 7 days of EGF infusion (Figure 3C and D) confirmed our mRNA data. In addition, DCX-positive cells appeared more dispersed, indicating less homophilic interaction between neuroblasts (Figure 3D).

### EGF induces a shift in the radixin population from DCX to Olig2 expression in the RMS

**Figure 4:**
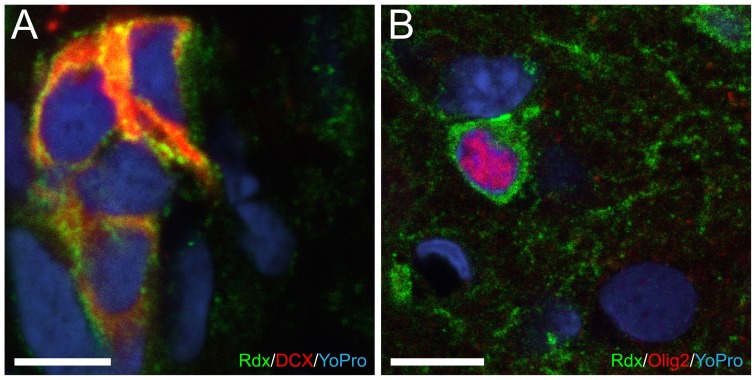
High magnification images of radixin^+^ cells. Immunofluorescence staining of cells coexpressing radixin(Rdx) and doublecortin(DCX) (**A**) or radixin and Olig2 (**B**) under control conditions. In (A) Radixin(green), DCX(red), and nuclear counterstain YoPro1(blue). In (B) Radixin(green), Olig2(red), and YoPro1(blue). Scale bar in A and B = 10 µm.

**Figure 5:**
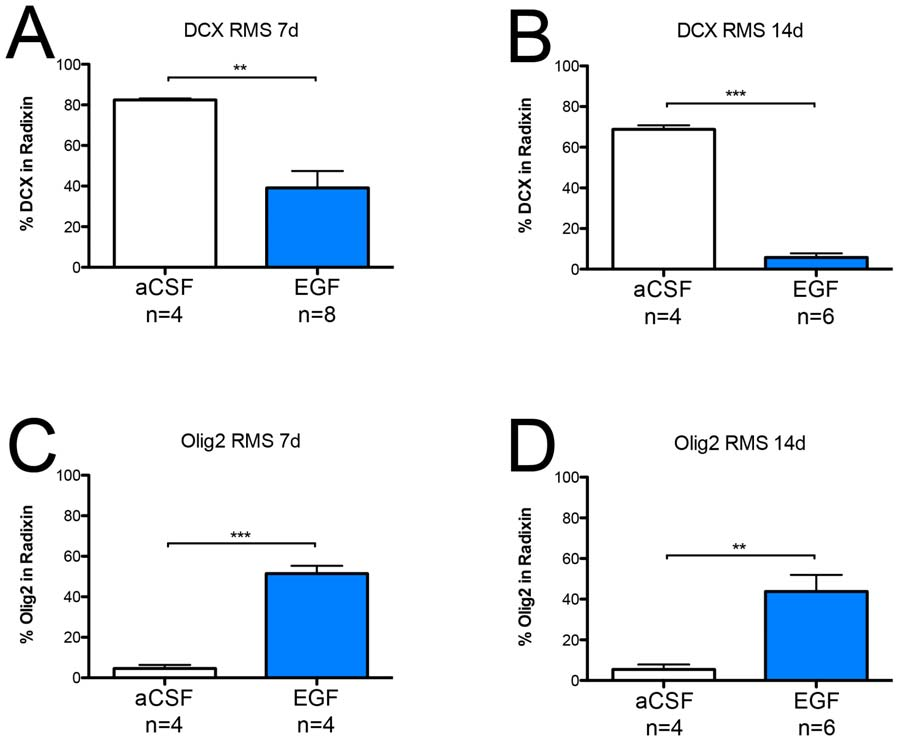
Olig2 and doublecortin expression in radixin^+^ cells. Graphs visualizing the percentage of cells (±SEM) expressing doublecortin (DCX) or Olig2 in radixin^+^ cells within the rostral migratory stream (RMS) (**A, C**) after 7 days and (**B, D**) after 14 days of EGF infusion. Statistical significance was assumed at ** p<0.01 and *** p<0.001 using Student's t-test.

Considering the association of radixin and DCX, we expected to see a similar downregulation of radixin expression. However, the relative mRNA level of *radixin* was upregulated two-fold in the SVZ (Figure 2C) and unchanged in the OB (Figure 2D) of EGF-treated animals. Using immunofluorescence, a large number of cells expressing radixin was detected in the RMS despite the decrease in DCX-positive neuroblasts; although the pattern of radixin expression appeared more dispersed and unorganized in the EGF-treated RMS (Figures 3 A, B and 5 A, B). We proceeded by analyzing the portion of DCX^+^ cells in the radixin^+^ cell population of the RMS (Figure 4A). Confirming our previous results, more than two thirds of the radixin expressing cells in the control RMS expressed DCX (Figure 5A and B). However, after 7 days of EGF infusion, the fraction of radixin-positive cells that expressed DCX was reduced to half of the control (Figure 5A, visualized in Figure 3E and F). The effect was even more pronounced after 14 days, where only 6% of the radixin cells expressed DCX (Figure 5B).

**Figure 6:**
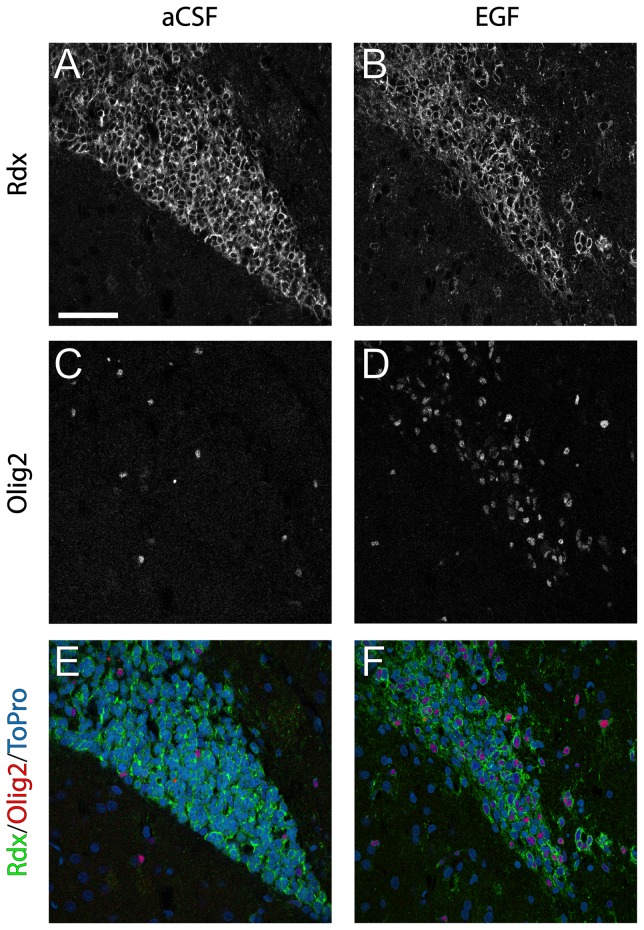
Radixin and Olig2 expression in the rostral migratory stream. Immunofluorescence staining illustrating radixin (Rdx) and Olig2 expression in coronal sections of the rostral migratory stream (RMS) after 7 days of aCSF or EGF infusion. (**A, B**) color separation for radixin, (**C, D**) color separation for Olig2, (**E, F**) color composite images of radixin (green), Olig2 (red) and the nuclear counterstain ToPro3 (blue). A notable increase in Olig2 positive cells is visible under EGF stimulation (**D**) compared to aCSF (**C**). The increased population of Olig2 positive cells expresses radixin (**F**). Scale bar in A = 50 µm.

We previously described a second population of radixin expressing cells in the adult brain, Olig2 positive cells, which are found throughout the brain, including in the RMS [14] (Figure 4B). After 7 days of EGF infusion, we detected a marked increase in Olig2^+^ cells, which co-expressed radixin (Figure 6). In control animals, the density of Olig2^+^ cells appeared similar in the RMS compared to the surrounding tissue. In contrast, radixin^+^/Olig2^+^ cells were highly enriched in the EGF-treated RMS. An 8–10 fold increase in radixin/Olig2 colabeling was found after 7 and 14 days of EGF infusion, when about half of all radixin^+^ cells in the RMS coexpressed Olig2 (Figure 5C and D). Since there was no difference in the radixin^+^/Olig2^+^ population between 7- and 14-day EGF infusions, we continued our analysis in the RMS of animals infused for 7 days.

### The EGF-expanded Olig2 expressing radixin cells are activated and enriched in the RMS

**Figure 7:**
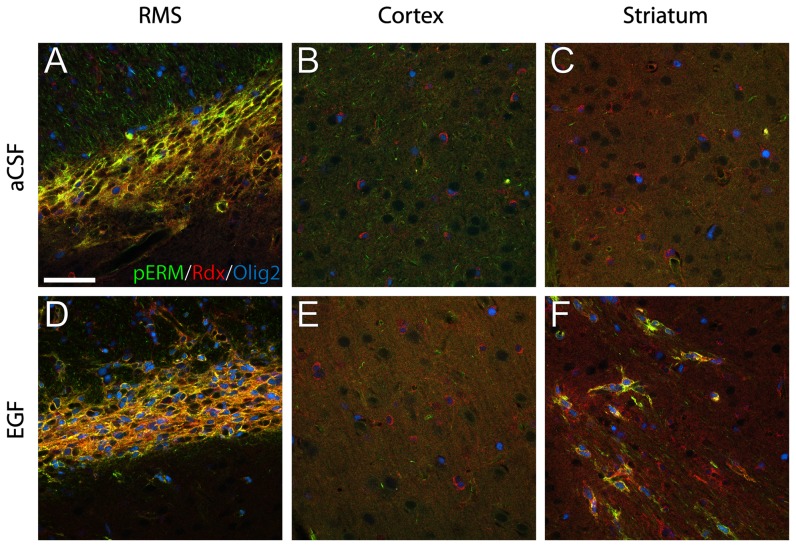
Phosphorylation of radixin in EGF-induced Olig2^+^ cells. Radixin (Rdx) activation was determined using an antibody that recognizes the phosphorylated form of ERM (ezrin, radixin, and moesin) proteins (pERM). (**A, D**) In the control rostral migratory stream (RMS), coexpression of radixin (red) and pERM (green) was predominantly observed in Olig2-negative neuroblasts. In the EGF-treated RMS the majority of radixin^+^/pERM^+^ cells was also Olig2^+^ (blue) cells. (**B, E**) No pERM expression was found in the cortex of either control or EGF-treated animals. (**C, F**) In the striatum, EGF infusion induced the presence of pERM^+^/radixin^+^/Olig2^+^ cells, which was not observed under control conditions (**C**). Scale bar in A = 50 µm.

To visualize activated radixin we colabeled cells with an antibody against phosphorylated ezrin/radixin/moesin (pERM). The radixin^+^ cells of the RMS were to a large extent positive for pERM, both in control and EGF-treated animals (Figure 7A and D). However, whereas in the control the radixin^+^/pERM^+^ cells were almost exclusively Olig2-negative, most radixin^+^/pERM^+^ cells expressed Olig2 in the EGF-treated RMS. Radixin^+^/Olig2^+^ cells outside the RMS were almost exclusively pERM negative (Figure 7B and E). Occasionally, radixin^+^/pERM^+^/Olig2^+^ cells with an elongated morphology were found in EGF-treated animals in the striatum bordering the SVZ and RMS (Figure 7C and F).

### Oligodendrocytes, astrocytes, and microglia in the RMS are unaffected, while Sox2-positive cells respond to EGF treatment

**Figure 8:**
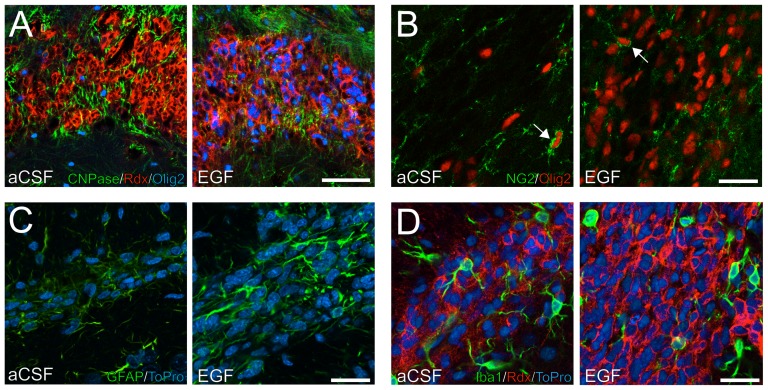
Expression of oligodendrocyte, astrocyte, and microglia markers in the rostral migratory stream. (**A, B**) Immunofluorescence staining against CNPase and NG2 show expression of oligodendrocyte lineage markers in the aCSF and EGF-treated rostral migratory stream (RMS). NG2^+^/Olig2^+^ cells were occasionally found under both conditions (arrows in B). (**C**) The appearance of the GFAP expressing cells was more reactive, with less regular and thicker processes, in the EGF-treated RMS compared to control. (**D**) Microglia were visualized based on Iba1 immunoreactivity. No differences between control and EGF-treated RMS were found in cell distribution or shape. Scale bar in A = 50 µm and B–D = 20 µm.

Previous reports indicate that EGFR activation can shift cell commitment towards the generation of oligodendrocyte-like progenitors [25], [26]. To determine if the RMS also contains EGF-responsive oligodendrocyte precursors, we investigated the expression of Olig2, NG2, and CNPase using confocal microscopy. Olig2-positive cells, both in the control and EGF-treated RMS, occasionally expressed CNPase (Figure 8A) or NG2 (Figure 8B). The overall CNPase and NG2 expression pattern appeared not to be altered by EGF (Figure 8A and B).

Astrocytes, which form the glial sheaths between chains of migrating cells in the RMS were analyzed by immunofluorescence against GFAP. Under EGF stimulation, GFAP expressing cells appeared less regular in shape with thicker processes, indicating a more reactive phenotype (Figure 8C).

Resident microglia are found throughout the brain, including in the RMS. Intrastriatal infusion of EGFR ligand TGFα in 6-OHDA lesioned animals was shown to increase Iba1-positive microglia in the SVZ and striatum [26]. However, no changes in the pattern of Iba1-positive microglia was observed in the RMS between the control and EGF-treated animals, and no Iba1^+^ cells coexpressing radixin were found (Figure 8D).

**Figure 9:**
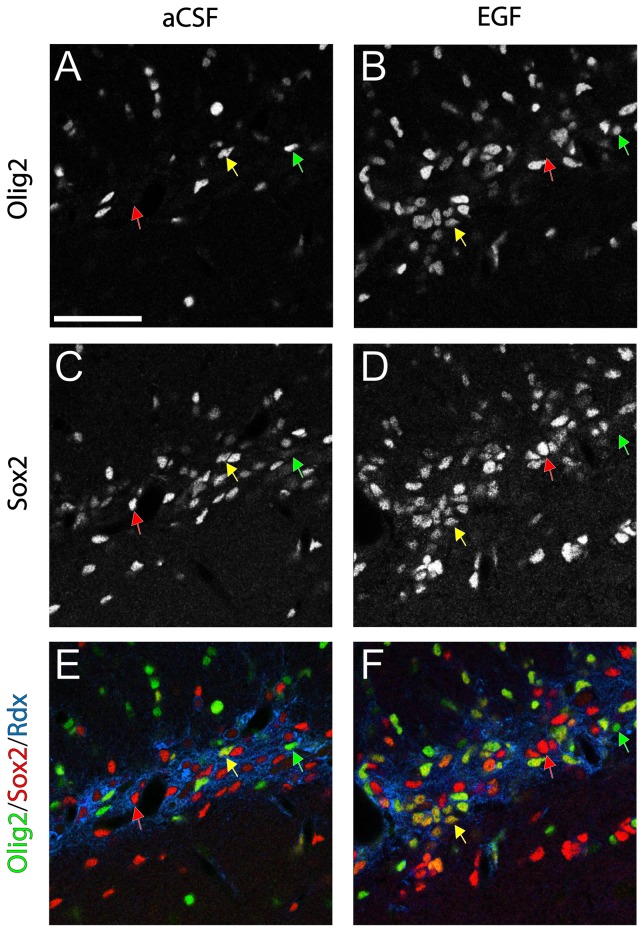
Olig2/Sox2/radixin co-expression in the rostral migratory stream. (**A, B**) Increased Olig2 immunoreactivity was observed after EGF infusion. (**C, D**) Olig2^−^/Sox2^high^ cells (red arrows) were found in both control and EGF-treated rostral migratory stream (RMS) (**A, C, E**) In the control RMS, Olig2 positive cells are few and either Sox2 negative (green arrows) or express Sox2^high^ (**B, D, F**) The expanded radixin^+^/Olig2^+^ population in the EGF-treated RMS express Sox2^high^ (yellow arrows). (**E, F**) Olig2^+^ cells negative for Sox2 were observed under both conditions (green arrows). Scale bar in A = 50 µm.

We previously reported that EGF induced hyperproliferation in the SVZ was largely due to an increase in Olig2 and Sox2 expressing cells [19]. These findings led us to investigate the expression of Sox2 in the radixin^+^/Olig2^+^ population of the RMS (Figure 9). In the control RMS, Sox2 was expressed in two separate populations, a low expressing population (Sox2^low^) that correspond to neuroblasts and a high expressing population (Sox2^high^) representing neural stem cells [27], [28]. In the control RMS, both Sox2^high^ and Sox2-negative cells were observed within the radixin^+^/Olig2^+^ population (Figure 9A, C and E). In the EGF-treated RMS, the EGF-induced radixin^+^/Olig2^+^ cells were predominately Sox2^high^ (73.1±4.97%, n = 5) (Figure 9B, D and F).

### The contribution of Olig2^+^ cells to the proliferating RMS population is increased after only 24 hours of EGF treatment

**Figure 10:**
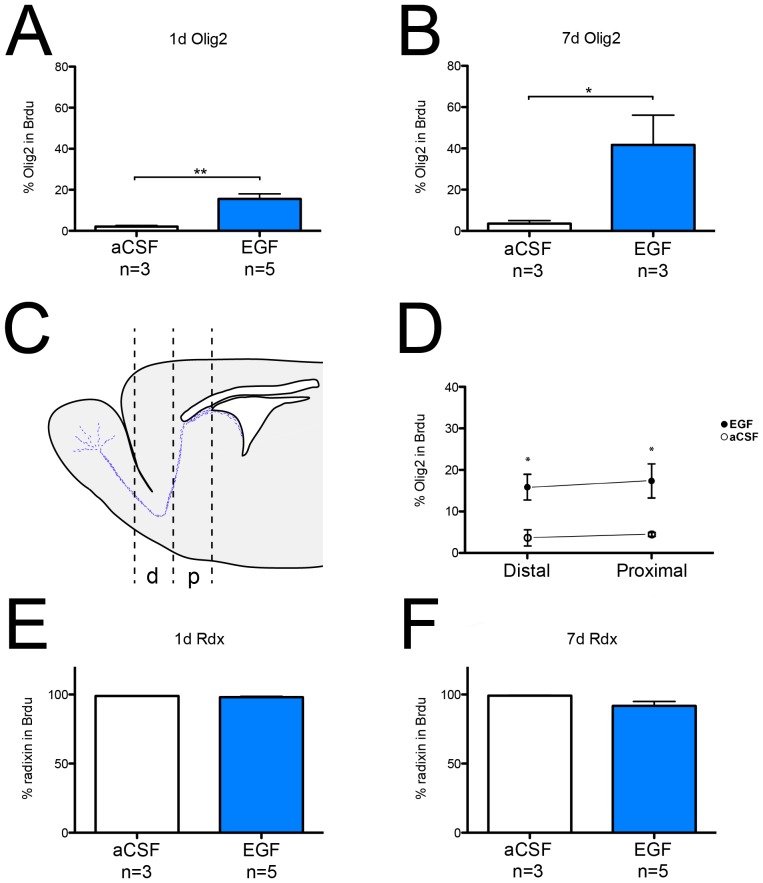
Proliferative response of Olig2^+^ cells in the rostral migratory stream to EGF infusion. (**A, B**) Quantification of BrdU/Olig2 co-labeled cells reveals an increase in the fraction of Olig2^+^ cells among the newly generated cells after 1 day (A) and 7 days (B) of EGF infusion. (**C**) Schematic illustration of the proximal (p) and distal (d) rostral migratory stream (RMS) coordinates used for analysis. (**D**) Quantification of the proximal and distal part of the RMS reveals a rapid response of Olig2-positive cells, at both locations, to EGF after 1 day of infusion. (**E, F**) Percentage of radixin (Rdx) expressing cells in newly generated cells (BrdU^+^) is close to 100% after 1 day (**E**) and 7 days (**F**) of aCSF and EGF infusion. Data are presented as percent of BrdU^+^ cells ±SEM. Statistical significance was assumed at * p<0.05 and ** p<0.01 using Student's *t*-test (A, B) or Mann-Whitney U-test (E, F). (**D**) n(control): proximal = 4, distal = 3; n(EGF): proximal = 5, distal = 4.

Considering the drastic increase in the fraction of Olig2^+^ cells expressing radixin, we studied the contribution of radixin and Olig2-expressing cells to the proliferative pool. We infused EGF for 1 or 7 days and administered three injections of BrdU during the last 24 hours before perfusion. The percentage of Olig2^+^ cells in the BrdU^+^ population increased substantially after 7 days of EGF infusion (Figure 10B). Surprisingly, already at 1 day of EGF infusion the portion of Olig2 expressing cells in the BrdU population was significantly increased (Figure 10A). To examine whether the responding cells are activated locally or originate from the SVZ, we then investigated the ratio of Olig2 expressing cells in the BrdU-labeled cells after 1 day of infusion at two positions along the anterio-posterior axis of the RMS (Figure 10C). The increase in the Olig2/BrdU ratio was observed in both the proximal and distal part of the RMS suggesting that the EGF-induced Olig2^+^ cells are derived from a local progenitor in the RMS (Figure 10D). This increase along the entire RMS was also evident after 7 days of EGF infusion (data not shown). At both time points, in both control and EGF-treated RMS, nearly all BrdU cells were also expressing radixin (Figure 10E and F).

### In vivo and in vitro chain formation suggest migratory properties of EGF-expanded RMS cells

**Figure 11:**
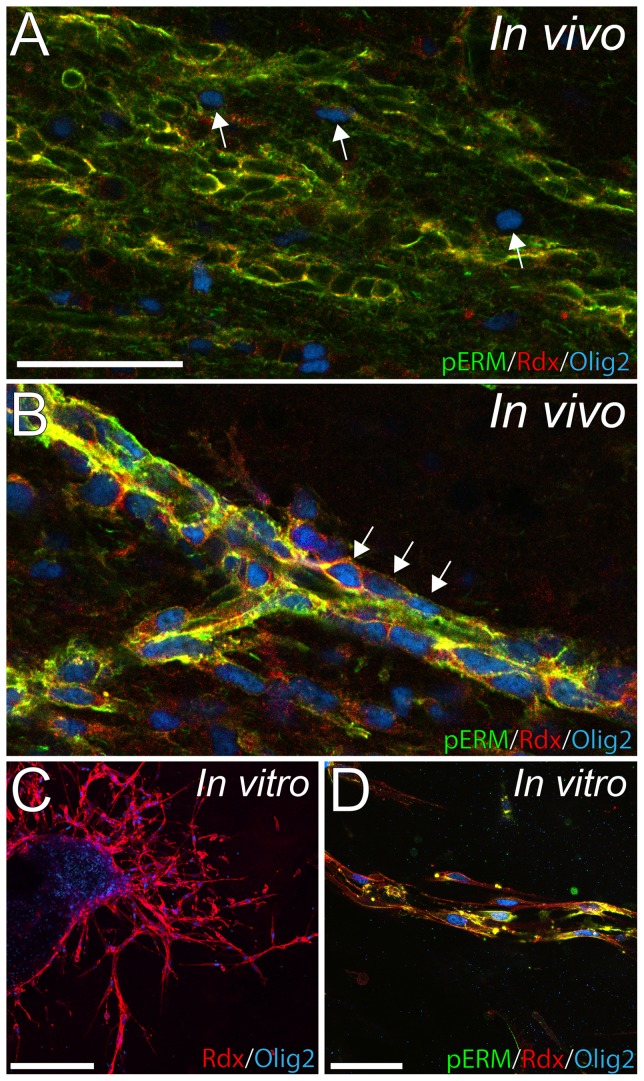
Alignment of Olig2^+^ cells in chains *in vivo* and *in vitro* explants. (**A**) Olig2^+^ cells in the control rostral migratory stream are few and separated (arrows). (**B**) Radixin (Rdx) and Olig2 co-expressing cells in the EGF-treated RMS express phosphorylated (ezrin, radixin, and moesin) (pERM) and are associated in chains (arrows). (**C**) Extensive radixin (red) and Olig2 (blue) immunoreactivity of migratory cells in explant cultures. (**D**) Tightly associated radixin^+^/Olig2^+^ cells in explant cultures resemble the appearance of *in vivo* migratory chains and also express pERM. Scale bar in A = 50 µm, C = 200 µm and D = 50 µm.

Frequently, and along the entire stretch of the EGF-treated RMS, we observed non-neuroblast cells lined up in chains. These cells were radixin^+^/Olig2^+^, tightly associated and had an elongated shape, similar to chain-migrating neuroblasts. In addition, chain-forming radixin^+^/Olig2^+^ cells and neuroblasts expressed pERM, unlike non-chain forming cells in both EGF and control RMS (Figure 11A and B). To study the migratory potential of the radixin^+^/Olig2^+^ cells, we used SVZ explant cultures. After 72 hours in culture, cells had migrated far from the explant core in tight chains. Numerous cells expressed βIII-tubulin or GFAP. Similar to our previously published data on neurospheres [14], radixin was expressed in explants in βIII-tubulin^+^ cells; however, co-labeling with GFAP^+^ cells could not be discerned (data not shown). The majority of migrating cells expressed radixin and a small portion was radixin^+^/Olig2^+^ (Figure 11C). These cells frequently formed chains and expressed pERM (Figure 11D).

## Discussion

In the current study we describe EGF-induced changes in radixin expressing cell populations in the RMS. The EGF stimulated RMS was less dense and displayed reduced DCX expression, while the number of radixin^+^ cells was unchanged. However, the phenotype of most radixin expressing cells shifted from a neuroblast to an Olig2 expressing progenitor cell. These radixin^+^/Olig2^+^ cells did not express oligodendrocyte markers CNPase or NG2, or the astrocyte marker GFAP, but were positive for the stem/progenitor cell marker Sox2. A strong increase in the relative amount of Olig2^+^ cells was observed in the RMS proliferative population after 7 days of EGF infusion. Moreover, a significant increase of Olig2-positive cells among recently generated (BrdU^+^) cells was measured in the entire RMS after just 24 hours of EGF infusion. The radixin^+^/Olig2^+^ cells in the control RMS were sparse, while EGF-induced radixin^+^/Olig2^+^ cells were numerous, occasionally formed migratory chains and showed activation in the form of pERM expression. Cells of the same phenotype displayed migratory properties and chain formation in explant cultures.

The RMS is highly proliferative, which is mostly attributed to rapidly dividing progenitors; however, the presence of slowly dividing stem cells has also been reported [15], [16]. Through intracerebroventricular infusion of EGF for 1, 7, and 14 days we were able to discern time-dependent EGF-induced changes in progenitor cells of the RMS. The rapid response of Olig2^+^ cells in the distal RMS after 1 day of EGF infusion speaks in favor of recruitment of local RMS cells, rather than migration of newly generated cells from the SVZ, which would require more time. BrdU labeling showed an increase in the portion of Olig2^+^ cells among the newly born cells at this location, which in turn indicates a local increase in proliferation within the distal RMS. The reduction in neuroblasts observed after 7 days was further enhanced after 14 days, while the increase detected in the radixin^+^/Olig2^+^ population plateaued after 7 days of EGF infusion. These data suggests that the generation of Olig2 expressing cells is a separate event from the decline in DCX expressing cells and not a direct fate shift.

Progenitor cells in the RMS react to EGF with increased production of Olig2 cells expressing Sox2, similar to their SVZ counterparts [19]. Increased Olig2/Sox2 coexpression can thus be observed in the SVZ, as well as the RMS after EGF treatment. EGF-induced Olig2^+^ cells can be reverted to neuronal fate determination after EGFR ligand withdrawal [26], [29], indicating that the fate shift towards a glial lineage is reversible.

Activation of ERM proteins by phosphorylation enables interaction with actin and transmembrane proteins resulting in cytoskeleton rearrangement. During tangential neuroblast migration the cytoskeleton needs continuous reorganization when protruding the leading process of the cell, and retracting the trailing end. In the control RMS, we observed, that radixin is phosphorylated in DCX^+^ neuroblasts. Radixin-positive cells co-expressing Olig2 were found throughout the brain. However, in these cells radixin appeared to be unphosphorylated. Under EGF stimulation, the pERM^+^ neuroblasts are lost; although the newly generated population of Olig2^+^ cells in the RMS now expresses phosphorylated radixin, suggesting a cytoskeletal activation in these cells. In addition, radixin^+^/Olig2^+^/pERM^+^ cells were more tightly organized than radixin^+^/Olig2^+^/pERM^−^ cells of the control RMS, and occasionally grouped together into chains. Similar cells were also observed in parts of the striatum of EGF-treated animals adjacent to the SVZ and RMS. Migration of Olig2^+^ progenitors after EGF stimulation has previously been described in the striatum [21], but not along the RMS. In our study, the ectopic migratory radixin^+^/Olig2^+^/pERM^+^ cells in the striatum were directed towards the RMS. Moreover, in explant cultures radixin^+^/Olig2^+^/pERM^+^ cells are migratory and can form chain-like structures *in vitro*. Although we present no direct evidence for a direct connection between radixin and EGF receptor activation, a possible link can be seen in the interaction between the EGFR and the ERM binding phosphoprotein 50 (EBP50), putatively modulating radixin function [30], [31]. EGF activation of the EGFR has been demonstrated to induce EBP50 expression *in vitro*. Conversely, increased expression of EBP50 has been shown to downregulate EGFR activity suggesting a negative feedback loop for EGFR by EBP50 [30], [32].

Previous studies infusing EGF into the adult brain have largely overlooked the effects in the RMS, except for the reduction in neuroblasts [17], [18]. The neural stem cell niche of the SVZ reacts to demyelinating injury and stroke with increased proliferation and migration towards damaged areas, however the SVZ is confined to an area deep within the brain. We report for the first time that locally recruited, putatively migratory progenitors in the RMS can be expanded by exogenous stimuli. Stretching across the entire forebrain, the RMS substantially increases the responsive zone that could be used in future brain repair and regeneration strategies.
